# Principles of Integrated Cognitive Training for Executive Attention: Application to an Instrumental Skill

**DOI:** 10.3389/fpsyg.2021.647749

**Published:** 2021-06-22

**Authors:** Francesco Benso, Sandra Moretti, Veronica Bellazzini, Eva Benso, Eleonora Ardu, Simone Gazzellini

**Affiliations:** ^1^Department of Psychology and Cognitive Sciences, University of Trento, Rovereto, Italy; ^2^ANCCRI, Associazione Neuroscienze Cognitive Clinica Ricerca e Intervento, Genova, Italy; ^3^AIDAI Liguria, Associazione Italiana per i Disturbi di Attenzione e Iperattività, Liguria, Italy; ^4^ASL 5, Azienda Sanitaria Locale, La Spezia, Italy; ^5^Department of Intensive and Robotic Neurorehabilitation, Bambino Gesù Children's Hospital Institute of Recovery and Care Caracterized by Research, Rome, Italy

**Keywords:** executive attention, working memory capacity, cognitive training, dyslexia, adaptive treatment

## Abstract

One effective cognitive treatment is the rehabilitation of working memory (WM) using an integrated approach that targets the “executive attention” system. Recent neuroscientific literature has revealed that treatment efficacy depends on the presence of various features, such as adaptivity, empathy, customization, avoidance of automatism and stereotypies, and alertness activation. Over the last two decades, an Integrated Cognitive Training (ICT) protocol has been proposed and developed; ICT takes the above-mentioned features and existing literature into account, and has been used to promote the development of reading skills. ICT has been employed in several clinical settings and involves stimulation of a specific deteriorated system (e.g., reading) and the improvement of executive attention components, thus also increasing working memory capacity. In this context, we present two experiments. In Experiment 1, participants diagnosed with dyslexia (aged between 8 and 14 years) underwent two ICT sessions a week, with home supplements, for a duration of 7 months. The participants showed a significant improvement in the reading speed of text, words, and non-words, and in the reading accuracy of text and non-words. In Experiment 2, we replicated Experiment 1, but included a comparison between two groups (experimental group vs. control group) of young participants with diagnosis of dyslexia. The experimental group was subjected to 18 ICT sessions twice a week and with home supplements, using the same protocol as in Experiment 1. The control group was entrusted to the protocol of compensatory tools and dispense/helping procedures provided by the scholastic Personalized Educational Plan. After training, the experimental group gained about 0.5 syllables per second in text reading, and a marked decrease in error rate. The control group showed no significant improvement in reading skills after the same period. Moreover, the improvement observed in the experimental group remained stable 4 months after ICT had ended. The results of these two experiments support the efficacy of the integrated ICT protocol in improving reading skills in children with dyslexia and its sustained effect.

## Introduction

One cognitive treatment that has been shown to be effective is an integrated approach that combines rehabilitation of working memory (WM) with that of the executive attention system. However, models and definitions of such theoretical constructs must be specified considering the numerous versions of the models introduced in neuropsychology over the decades, and the evolution of each model over time. The present study aimed to add to the knowledge on cognitive treatments by highlighting crucial points raised in the existing literature, and thus to increase the efficacy of cognitive treatments.

### Working Memory, Executive Attention, and Attentional States

The expression “working memory” can be easily misunderstood due to changes and improvements to the initial models of WM, which have evolved alongside neuroscientific discoveries in the field. This evolution has been mainly characterized by a clearer definition of the executive control system. Indeed, the executive control system was initially described rather vaguely as a “central processor” (e.g., Baddeley and Hitch, 1974), but, over time, it acquired a clearer evidence-based framework. WM models have therefore changed over time, and have moved toward a greater convergence (e.g., Baddeley and Hitch, 1974; Cowan, 1988; Engle et al., 1999; Cowan et al., 2002). The more widely shared and clearly defined system in this theoretical evolution is the central executive, which is a multicomponent system that includes several known and unknown executive-attentive functions (Engle and Kane, 2004; Repovš and Baddeley, 2006; Hofmann et al., 2011).

In this context, recent neuroscientific and imaging evidence has led researchers to consider cognitive functions as an expression of cortical-subcortical networks that are subjected to several activation states, rather than being the result of a single cortical area that is measurable using a single test (Bernstein and Waber, 2007; D'Esposito, 2007; D'Esposito and Postle, 2015). Therefore, we are in favor of more general and less reductionist theories, such as the Executive Attention Model (Posner and Di Girolamo, 1998; Engle et al., 1999; Rueda et al., 2005; McCabe et al., 2010; Hofmann et al., 2011), which, according to some authors, expresses in working memory capacity (WMC; Engle and Kane, 2002). The executive attention system involves a synergy between executive, attentive, and memory processing (Repovš and Baddeley, 2006). This system is primarily aimed at processing, maintaining, and reworking any information that is relevant to the ongoing task, and discarding irrelevant information. Among the components of executive attention, goal maintenance and competition resolution are the most strongly associated with WMC. These components involve several attentive and executive functions that are similar to those of the WMC model by Cowan and colleagues (Cowan, 1988; Cowan et al., 2002). Cowan's model is characterized by the almost exclusive inclusion of attentive executive components, i.e., the focus of attention directed toward long-term memory that is supported by resources of the central executive system. The other important WM model of Baddeley (1986) has also been modified to become more similar to the WMC model (e.g., Engle and Kane, 2004; Hofmann et al., 2011; for a review on WM model evolution, see D'Esposito and Postle, 2015).

These models have the advantage of combining attentive, mnestic, and executive aspects, thus avoiding extreme and impractical reductionism that aims to isolate and erroneously measure single executive functions (Rabbitt, 1997; Repovš and Baddeley, 2006; McCabe et al., 2010; Benso, 2018). Mounting evidence supports the notion that the learning of complex motor and cognitive skills during development largely involves executive attentive circuits that include the anterior cingulate cortex, dorsolateral prefrontal cortex (DLPFC), intraparietal cerebral sulcus, and basal ganglia (Engle and Kane, 2002, 2004; Sakai et al., 2002; McNab and Klingberg, 2008; Petersen and Posner, 2012; Seidler et al., 2012; Leisman et al., 2014). The literature on training of executive attention and, by extension, WMC has indicated that training induces macrostructural brain changes. Olesen et al. (2004) have reported the changes in WMC-associated brain activity after cognitive treatment, whereby an increase of this activity was observed in the medial frontal gyrus and in the inferior and superior parietal cortex. Takeuchi et al. (2010a,b, 2011) have highlighted cognitive changes and modifications in brain connectivity and gray matter thickness following cognitive training. Other authors have reported changes in microstructural and neuromodulation following training; for example McNab et al. (2009) showed that an increase in WMC is linked to a change in cortical density of D1 dopamine receptors.

### Terminology and Systems Development

It is necessary to clarify terms that have emerged from a series of converging, but not always similar, theories. As explained in section Working Memory, Executive Attention, and Attentional States, the terms WM and WMC converge in many ways, despite the fact that the originally proposed models were quite different (e.g., Hofmann et al., 2011). We will use both the terms WM and WMC, according to the definition of Hofmann and coll., provided that they reflect the models previous explained. WMC differs from short-term memory, and has instead been proposed to contain short-term memory as a subsystem (Engle and Kane, 2004). D'Esposito et al. (2000) have reported that brain circuits underlying WM, which include the DLPFC at their epicenter, are not activated during a simple span task, while a strong activation appears whenever there is material that needs to be updated. We will refer, in this sense, to more or less activated brain circuits, excluding outdated and tempered models that recall specific warehouses and areas (e.g., D'Esposito, 2007; Morra, 2011). WMC is fed by a multicomponential attentional and executive system that subserves a range of complex functions and information reworking processes, while also maintaining the current goal as active, despite interference from distractors. The WMC system is anatomically and functionally connected to long-term storing networks and to peripheral sensory networks (Duncan and Owen, 2000; Jonides et al., 2005; D'Esposito, 2007). As already noted, there have been some misunderstandings about the definition of WM due to the different meanings conferred by WM models over the years. Morra et al. (2018) maintain that WM has previously been incorrectly (and in a reductionist way) used to describe one of the several executive functions, particularly when related to updating (e.g., Miyake et al., 2000). The authors have described these theoretical-methodological misunderstandings as follows: “*Incidentally, we note that if one accepts the componential theory of working memory, it would be illogical to claim that working memory is an executive function. This is because asserting that working memory includes a central executive, the central executive includes the executive functions, and the executive functions include working memory is clearly a vicious circle*” (Morra et al., 2018, p. 250).

However, it is worth remembering that the WM system develops alongside neurocognitive maturation. Crone et al. (2006) reported that the cortical areas for re-updating in working memory, and which are centered on the DLPFC, are not frequently recruited before the age of 8 years. Furthermore, Morra (2015) maintains that the rehearsal process is not completely active before the age of 7 years. Bunge and Zelazo (2006) have demonstrated that abstract reasoning and complex switching skills develop after the age of 6–7 years. Other relevant studies are those that have investigated the Theory of Constructive Operators [Pascual-Leone and Goodman, 1979; see also Panesi and Morra (2017, 2020)]. As shown by Giedd (2015), development of the WM system and Central Executive Network (CEN; see section Attentional Networks) ends many years after infancy. During adolescence, executive and WM maturation slows down in favor of the limbic system for emotion control, but their development restarts at the age of around 18–20 years and continues for another decade, from the ages of 20–30 years.

As far as networks are concerned, which are described in more detail in section Attentional Networks, Rueda et al. (2005) noted the development of a combined stimulus-driven and top-down attention control in the 1st months of life. Rothbart et al. (2011) proved CEN activity in 24 month-old children. An anti-correlation between the CEN and Default Mode Network (DMN) was observed by Gao and Lin (2012) in 12 month-old children. The Salience Network (SN) can be readily identified by the of age 2 years, and it undergoes protracted changes in connection strength throughout childhood (Gao et al., 2013). Between the ages of 7 and 20 years, the SN, CEN, and DMN undergo further developmental changes that involve both within- and across-network links (Uddin et al., 2011).

### Attentional Networks

Defining attentional states and the associated neural networks and anatomical models is crucial to understanding the principles of cognitive treatment. The neuroscientific literature has documented the presence of several neural networks underlying attention. Some of these have long been known (Posner and Petersen, 1990), such as the networks underlying phasic and tonic alert, automatic and voluntary attention orienting, and the CEN of the first executive control system. The CEN actually includes the anatomical substrate of executive attention and WMC (Engle and Kane, 2002). The CEN comprises the anterior cingulate cortex, and dorsolateral prefrontal and parietal cortices; it exhibits a subcortical coupling, and is engaged in higher-order cognitive and attentional control (e.g., Menon and Uddin, 2010).

Raichle et al. (2001) described a functional-connected brain network comprised of the precuneus/posterior cingulate cortex, medial prefrontal cortex, and medial, lateral, and inferior parietal cortex (the DMN), which is active during resting states. Further studies have described several DMN functions, such as attentional shifting from the external task (lapses of attention), mentalization, introspective thinking, and mental simulation (Fransson, 2005, 2006; Buckner et al., 2008; Fassbender et al., 2009; Andrews-Hanna, 2012; Gazzellini et al., 2017). During the performance of cognitively demanding tasks, the CEN typically shows an increase in activation, whereas the DMN shows a decrease in activation; in that sense, the two networks are anti-correlated (Dosenbach et al., 2008; Menon and Uddin, 2010).

The third crucial attentive network is the SN, which includes the anterior insular and anterior cingulate cortices, and has extensive connectivity with subcortical and limbic structures involved in reward and motivation. The SN is essential for monitoring external input saliency and internal brain events (e.g., Sridharan et al., 2008). In particular, the SN and anterior insula play a critical and causal role in switching between the frontoparietal CEN and the DMN across task paradigms and stimulus modalities, which suggests a causal and potentially critical role of the anterior insula in cognitive control (Dosenbach et al., 2008; Menon and Uddin, 2010). Moreover, Sadaghiani et al. (2010) reported that tonic alertness is maintained by the activity of areas belonging to the SN. Indeed, spontaneous fluctuations of alpha oscillation power have been found to be correlated with activity in the cingulo-opercular network (including the dorsal anterior cingulate cortex, frontal operculum/anterior insula, and thalamus), which has been related to sustained cognitive control and alertness maintenance (Dosenbach et al., 2008; Sadaghiani et al., 2010). These studies clearly reveal the cerebral systems' complexity, although some of the literature goes on to describe hypothetical and specific functions, which were investigated using single tasks and associated with distinct cortical areas (Bernstein and Waber, 2007). In the view of a complex cerebral system as function of dynamic neural network interactions, failure in an executive-attention test no longer implies a CEN weakness. On the contrary, there are at least four alternative hypotheses, as follows: 1. CEN weakness, 2. The SN does not correctly activate the CEN, 3. DMN activity dysfunctionally intrudes during CEN activity, which breaks the DMN-CEN anti-correlation rule, and 4. A dysfunctional interaction among the three circuits may cause a decrease in executive-attention task performance (see Uddin, 2015).

In other words, it is worth underlining that the interaction between these attentional networks has increased the explanation complexity of functional deficits and has even changed the anatomo-functional models of some neurological pathologies (Menon, 2015; Uddin, 2015). The functional network models described are crucial in the study of cognitive treatment, as proposed in this manuscript. We have selected cognitive tasks that engage such attentional networks, with the aim of improving our assessment protocols and intervention measures such that they are in agreement with the up-to-date knowledge on interactions between the SN, CEN, and DMN (see also Uddin, 2015).

Work by Petersen and Posner (2012) and Tang et al. (2012) has highlighted the neural networks underlying orienting of attention and phasic alert, even though these authors have proposed models that conflict somewhat. Corbetta and Shulman (2002) proposed that a noradrenergic circuit comprising the locus coeruleus, temporo-parietal junction, and fronto-ventral cortex is related to the system of automatic orienting of attention and to external stimuli detection. The existence of such a network has been confirmed by Kucyi et al. (2012), but contrasting data are also available (see Geng and Vossel, 2013). Boundaries and switching between physiological arousal, phasic alert, and tonic alert have been described differently by several authors, as have the definitions of sustained attention and alertness (Sturm and Willmes, 2001; Sadaghiani et al., 2010; Petersen and Posner, 2012). However, to develop the aim of the present study, we adopted the definitions by Sturm and Willmes, as follows: “*Phasic alertness is required whenever a warning stimulus in the same or a different sensory modality precedes the target stimulus. Sustained attention in contrast to alertness tasks typically do not focus on pure speed of response but rather on the number of hits, misses (and false alarms), and their time course*” (Sturm and Willmes, 2001; pp. 76–77). Studies on phasic alert (see Tang et al., 2012 for its innateness) have been pivotal in developing treatment protocols; indeed, their findings have been employed to overcome low tonic alertness, which must be reinforced and treated delicately in patients with weak sustained attention. The shift from phasic to tonic alert and activation exercises are important in the training protocol we propose in this paper. Around 20 years ago, one of the present authors studied the interaction between the focusing of attention and warning curves (Benso et al., 1998) and the different warning styles during attentional shifts between audition and vision (Turatto et al., 2002). These studies and the related scientific literature led to proposals of training techniques that require participants' cognitive activation before the rehabilitation or study sessions, and which also allow weak sustained attention to be studied without requiring exercises that directly affect sustained attention, instead using a progression of warning states (Benso, 2004a). Indeed, this progression of warning states is one of the most important elements of our proposed training.

### Specifications of Cognitive Treatment

Cognitive treatments usually include different types of exercises, ranging from the numerous downloadable internet applications to logical games, or from “pencil and paper” exercises to virtual reality and video tutorials on attention and WM, among others (Cancer et al., 2020; for a review, see Galuschka et al., 2020). Thus, the term “cognitive treatment” is an umbrella term that fails to communicate precise or shareable information if the rehabilitative principles are not considered. This aspect is not insignificant, particularly if we consider the frequency with which the term “cognitive treatment” is used in the literature.

That said, the variability of approaches seems to be common, even within a single treatment. Indeed, previous meta-analyses have often failed to provide clear and shareable results. This is probably due to the large number of variables involved in the studies, which are often uncontrollable. In this sense, it is notable that even though meta-analyses have been largely limited to WM treatments, there are still large disparities in previous findings that have resulted in conflicting opinions, particularly around the aspects of transfer possibilities, the type of treatment chosen in the analyses, and the methodology used (Melby-Lervag and Hulme, 2013; Au et al., 2014; Karbach and Verhaeghen, 2014; Schwaighofer et al., 2015; Melby-Lervåg et al., 2016). As a consequence, recent work has attempted to clarify the topic by identifying intervening variables that are crucial for treatment goodness, and which can be easily neglected during treatment administration.

An intriguing example comes from a neuroimaging study on plasticity. Metzler-Baddeley et al. (2016) write the following: “*Evidence for training-induced brain plasticity remains inconsistent and controversial (Thomas and Baker*, *2012**). Longitudinal studies into macrostructural brain plasticity have found mixed findings regarding the direction of training-induced alterations in gray matter volume or cortical thickness in task-relevant brain regions. For instance, Draganski et al. (**2004**) found increases in gray matter volume in temporal and parietal regions after 3 months of juggling whilst, Takeuchi et al. (**2011**) reported reductions of gray matter volume in parieto-frontal cortical regions after working memory training*” (p. 48). It is therefore necessary to take into account, as much as is possible, the different variables that affect behavior and plasticity. These include the brain area involved, subjects' age, training duration, administration time, exercise intensity, treatment type, the setting in which treatment is carried out, calibration and difficulty of the exercises, the presence of a skilled operator who is able to motivate participants and regulate emotions (Schubert et al., 2014; Schwaighofer et al., 2015).

When designing rehabilitation protocols, it is essential to consider all these variables, and to clearly define the term “adaptive treatment.” Training is “adaptive” when the operator or software takes participant's errors and decreases in reaction times into account, and readily recalibrates the material according to a subject's performance potential to ensure that the training is productive (in that, there is no progress with exercises that are too easy or too difficult). A training is “not adaptive” when the skills required greatly exceed or are inferior to the actual skills of a subject. In this case, the subject remains in an automatic and stereotyped mode for too long. Without attentive awareness, these repetitive exercises can lead to boredom, distraction, and the creation of rigid and closed patterns; even if improvements are achieved, they tend to be unstable over time or cannot be generalized to new contexts. The time spent in automatic or stereotyped mode is a crucial intervening variable that operators should take into account. High levels of non-adaptivity may transform a treatment into a pseudo-treatment, but both may be erroneously classified or analyzed as treatments, particularly in meta-analyses of effect sizes.

Unfortunately, the need to overcome automatism during the treatment and learning phases is rarely acknowledged, even by experts^1^. To examine this aspect and its consequences (in that, strictly speaking, “non-adaptive” training should not even be defined as “training”), we can refer to work by Metzler-Baddeley et al. (2016). In that study, performance was compared between an experimental and a control group. The experimental group was subjected to an “adaptive” treatment that involved a gradual increase in complexity, which was calibrated for each individual participant. The control group was subjected to a “non-adaptive” treatment, which had a low difficulty and involved repetitive tasks. Neuroimaging revealed that the thickness variation of crucial brain areas was in the opposite direction between the two groups. The authors conclude that these results indicate that the direction of activity-induced plastic changes is influenced by the complexity of training, as well as brain location (Metzler-Baddeley et al., 2016; p. 48).

These results indicate that adaptivity is an important variable to take into account; indeed, the effect of adaptivity seems to have been reported in very different research fields, such as that on memory prediction (Wagner et al., 1998). Wagner et al. found that when frontal-lateral areas are not activated during a task (including the DLPFC, which is an important hub of the neural network supporting the different functions of “executive attention”; Engle and Kane, 2002, 2004), learning is not consolidated, and the experimenter can even use neuroimages to predict the words that will be recalled or forgotten by the subject. This evidence can clearly be transferred to the context of rehabilitation, and several authors have suggested ways to activate these areas during treatment. To this aim, D'Esposito et al. (2000) have pointed out that cortical networks that include the DLPFC are activated during specific exercises that engage the “executive attention” system (Engle and Kane, 2002), such as the n-back task, dual task, and update in working memory tasks.

As suggested by Rothbart et al. (2011), when children develop cognitive skills, minor activations occur in prefrontal areas, which could indicate a less dispersed and more specialized tuning of neurons. These authors have also argued that similar aspects can be detected in adult learning. During learning, the number and size of brain activations are reduced (Kelly and Garavan, 2005), and a significant increase in brain connections occurs with practice (Fair et al., 2009). To increase training efficacy, we must understand how to activate CEN circuits that include the DLPFC using WMC tasks and to involve executive attention (e.g., using the reworking in memory task, n-back task, and dual-tasks). Moreover, exercises involving complex motor learning have been found to show the same training effect (Sakai et al., 2002; Badre and D'Esposito, 2007; Seidler et al., 2012; Leisman et al., 2016; Benso, 2018). In some cases, a transfer of improvement to instrumental skills (i.e., reading, computation, and IQ) has been shown, albeit with a medium/low effect size (Klingberg et al., 2005; Dahlin, 2010; Loosli et al., 2011).

The above literature reinforces the idea that stereotypies and automatisms should be avoided during training, and that it could be helpful to involve circuits that include the DLPFC during the rehabilitation process, since these elements are necessary in the acquisition phase. This evidence helps to establish the essential criteria of our Integrated Cognitive Treatment (ICT) of executive attention. ICT protocols have already been applied to patients with head trauma, degenerative diseases, children with developmental disorders, and athletes, as well as to develop learning procedures in the classroom (Benso, 2018). In the following sections we will describe the treatment criteria, the protocol, and two studies of the efficacy of ICT on reading skills in children.

### Essential Criteria of Integrated Cognitive Treatment

Many of the work described in the previous sections have allowed us to identify fundamental and essential elements that are required to create an effective treatment protocol, and which thus constitute the ICT structure. The essential ICT criteria identified are as follows:

a. Considering what cognitive neuroscience has revealed about the theoretical models of learning, different aspects of attention and some executive functions are expressed in WMC. Moreover, it is necessary to understand the functional architectures to integrate the underlying trigger to the servo-systems of deteriorated or underdeveloped modular circuits.
b. An empathic and motivating operator administering the treatment, who can also control computer software (Schwaighofer et al., 2015).
c. A trainer who has the following skills: (i) the ability to vary and recognize attentional states (with targeted exercises that aim to activate, maintain, change, and strengthen each state), and understanding the different levels of attention (which allows for the creation of optimal “flow states” during learning and enables the phase of activation; see section Attentional Networks; Csikszentmihalyi, 1996; Gazzellini et al., 2016); (ii) The capacity for empathic bonding with the trainee; and (iii) the ability to calibrate items and exercise difficulty according to the skills of individual subjects, and to carefully evaluate the development of skills during the training (adaptivity of the training). Trainers should also avoid interventions that are involve repetitive, stereotyped, and automated loops.

During the treatment course, the ability to take the person back (as much as possible) in a required setting through conscious “here and now” activities and provide immediate feedback. This provides the necessary repetitions that are aimed at creating hyper-learning and expertise, and avoid stereotypical settings.

ICT requires these points to be satisfied. Moreover, skilled supervisors play a fundamental role in ICT. Current treatment proposals tend to focus on the instruments of the treatment, such as the increasingly sophisticated automated technologies and software. We recognize their usefulness, particularly when subjects cannot be treated directly and continuously. However, increasing evidence shows that a “human” presence can further enhance the effects of rehabilitation (see Schwaighofer et al., 2015). In ICT, a supervisor acts as an empathic “motivator,” careful observer, and trigger of attentional and emotional states by implementing exercises that engage the SN, CEN, and DMN. The supervisor also plays a crucial role during the sessions that precede treatment, during which they define and tailor exercise settings according to the functional levels of the specific person to avoid non-adaptivity of the treatment.

ICT is therefore an adaptive rehabilitative treatment. It is defined as integrated because, it aims to strengthen not only the attentive-executive system, but also specific systems that are underdeveloped or deteriorated (reading, for example) and their underlying functions (e.g., language and visual-perception). In line with the research already described, the theoretical framework we refer to Benso (2004a,b); Benso (2018) argues that all types of learning, even of minimal complexity, require attentional resources. Therefore, a rehabilitative intervention for a specific function cannot deal only with the deteriorated specific module, but must also be extended to the attentive executive system, which is required for the modularization process (see Karmiloff-Smith, 1992).

As described above, one of the essential elements of ICT fundamentals is that the subject has to be activated and carried in an attentive set (see Fuster and Bressler, 2015), hence motivated and guided by the supervisor, before starting the actual treatment. This is realized by referring to different attentive theories (see section Specifications of Cognitive Treatment), such as automatic orientation and alert phase, which are innate systems that should be activated and trained (Tang et al., 2012). These activities use very simple cognitive and psychomotor tools and are based on the subject's existing skills. In the pre-training phase (2–3 min before the training), “Ready-Go” activation tasks are implemented, such as ball throwing, gesture imitation, dual task naming, and rapid card picture recognition, and the stimulus presentation frequency is tuned to the subject's response level. In the second training phase, these exercises are employed to prolong the state of alertness and to induce phasic to tonic switches, which acts to promote the participant's sustained attention during the training tasks. Hence, sustained attention is increased by strengthening participants' innate phasic alert system, which avoids directly pointing out their weaknesses and leads to the paradox of asking an inattentive person to sustain his/her attention. Therefore, the ICT protocol includes initial activation tasks that trigger the warning system in phasic alertness (Benso, 2004a). Subsequently, in the course of the treatment session, sustaining exercises are implemented, such as dual task, updating in working memory, executive control of conflict, cognitive flexibility, self-regulation through different sensory modes, and mental images to be processed.

The ICT protocol has been customized and applied to amnesic people with mild cognitive impairment, and its effects evaluated by neuropsychological and positron emission tomography (PET) measures (18F-FDG-PET; Ciarmiello et al., 2015). In that study, the experimental group was treated for 4 months with ICT, and the control group received psychological support at the same time. The pre-post treatment comparison revealed that significant changes in brain metabolism had occurred in the cortical areas involved in WM and attentive-executive processing, and these changes were associated with improvements measured in the neuropsychological assessment. Further studies have supported the efficacy and validity of ICT in people of various ages and pathologies, including older people, adults with head trauma (Benso, 2004a,b), and in children with developmental delay (e.g., dyslexia, Benso et al., 2008; Benso, 2010). For example, ICT has been found to result in significant improvements in executive attentional and academic tests with respect to controls in children (Veneroso et al., 2016, 2018), and a better performance in executive attentional tests compared with controls in athletes (e.g., tennis players, Benso et al., 2018).

### ICT: Exercises and Materials

In the opening phase (pre-training) of ICT, there is first a harmonization of the subject conditions (physiological, attentional, emotional, and motivational conditions). Second, there is an enhancement of executive cognitive attentional circuits (which is also useful for emotional self-regulation). In a final third phase, ICT can be specifically applied to weak systems (such as reading, writing, and computation), those with neurological pathologies, older people, or on sporting/artistic abilities. The last two phases can be integrated, and certain exercises can be shared and tailored according to the type and severity of a pathology and age of the patient (also considering didactic needs and sports specialty). In the present study, the chosen third-phase treatment was reading. Many of the exercises have also been translated into “cognitive motor activities.”

#### The Opening Phase: Physiological Arousal and Attentive Activation

The conditions of the subjects are carefully considered before starting treatment, since it is possible that subjects are not willing to cooperate. For example, children who arrive in the clinical setting in the mid-afternoon, after school hours, might be demotivated, emotionally discharged, distracted, tired, or over-excited. Therefore, in the first 5–10 min, attempts are made to normalize emotional, motivational, attentional, and psychophysical arousal to prepare for the cognitive tasks that follow, which are tailored to the participant.

According to the Hebb/Yerkes-Dobson curve, the arousal state should be “centered.” To achieve this, arousal should be activated when it is too low (in this case using the motor system, with simple imitation or naming exercises that are calibrated to the subject) or calmed down when it is too high (by breathing exercises combined with coordinated and slow gestures, or tasks requiring concentration on a particular sound vibration, as occurs in some Zen meditation schools)^2^.

In the meantime, the subject can become motivated by receiving clarification of the purposes of the session, and emotional states addressed by creating an empathic relationship, whereby the operator welcomes the child, and demonstrates solidarity with their discomfort and weaknesses. Any chronic emotional disturbance is treated separately.

We will now describe the training phases, exercises, and materials used in the ICT protocol, which were tailored to create integrated executive attention-reading training. It is worth underlining that the material itself is interchangeable and can be altered according to the specific needs of a subject; the crucial element is instead the nature of the exercises, which aim to elicit different attentional states that are maintained over time, and to enhance the resources of executive attention and WMC.

##### Opening Phase

a) A fast naming (Rapid Automatized Naming task: RAN) task using interference stimuli (numbers, colors, letters, and/or shapes at the same time) on cards and scoreboards, as well as concurrent calibrated tasks (verbal or motor) and copying simple gestures.

b) Breathing exercises combined with coordinated, slow gestures.

##### Executive Attention and Reading Training Phase

a) Potential cards: Updating and divided attention training, multi-tasking processes, shifting activities and executive control with multimodal stimulations, including verbal (fast naming) and motor (gesture) tasks.

b) Matrices: Matrices are grids (whose measures have been tailored to each person) with different stimuli (numbers, letters, shapes and/or empty cells). Visual and auditory updating, spatial orientations, generation of mental images, and sustained and focused attention tasks were completed, showing positive effects also on the method of study (Benso, 2011). The task required participants to store the information presented in horizontal rows of a matrix in long-term memory, and to generate a mental image. Then, participants were requested to maintain and rework these stimuli in different orientations (horizontally, vertically, diagonally, and with rotation) by manipulating the mental image generated. Symbols inside the matrix change according to the subject's profile and can also be motor sequences or paths to be repeated, optimal in sports sets and hyperactive subjects.

c) The Attention and Executive Processing-10 min (APE 10): Visual and auditory training that in just 10 min triggers different attentive executive functions using cards and scoreboards with n-back tasks. This exercise provides parallel stimulation of the visual-perceptual aspects involved in reading, including attentive focus, crowding and contrast effects, and ocular movements of pursuit and saccades (Benso, 2011). The APE 10 is an original measure taken from the Paced Auditory Serial Addition Task (PASAT) test (Gronwall, 1977), which includes serial addition and multiplication tasks (n-back task). Other measures such as the Paced Auditory Serial Opposites Task (PASOT, Gow and Deary, 2004) avoid numbers with n-backs. It is also possible to transform the task into a kind of speech therapy by using letters or syllables that merge into words.

##### Specific Treatment of the Underdeveloped Module (Reading)

a) Spoonerisms (swopping initial phonemes between two words) and metaphonological awareness exercises (phonemic and syllabic fusion and segmentation; Benso, 2011) were applied in ICT. However, to establish these skills, there must be an adequate updating capacity in WM.

b) Tachistoscope and turbo-reader “LEGgo” in the ICT application to reading skills was used. This tool provides reading exercises, from sub-lexical aspects to disappearing text. It was designed to train meta-phonology, with fusion and segmentation exercises, as well as treatment of the crowding effect, and has many linguistic and visuo-spatial aspects that play a role in reading, such as letter-span (attentional focus width, Zoubrinetzky et al., 2019) and abnormal ocular movements (exposure times are modified and take saccadic eye movements and visual persistence into account; Coltheart, 1980; Benso, 2010; also see section Application of ICT to Improve Reading Skills). All the exercises are administered with a timing pressure that is tailored to the participant's skills, which triggers the attentive networks during task execution.

Each exercise was tailored to each participant and to ensure an adaptive treatment. This calibration was performed by a skilled supervisor at the beginning and during the training. The material was recalibrated according to the actual abilities of the subject to ensure that the training was productive (there is no progress with exercises that are too easy or too difficult). The ICT protocol has also been adapted to other different integrated systems, such as executive attention and writing, computation, problem solving, motor control, motor learning, and visuo-spatial processing.

### Application of ICT to Improve Reading Skills

Reading is considered to be a multi-componential process. Findings in the field of developmental dyslexia have often been conflicting, and some issues remain controversial. For example, among the theories on the processing of linguistic structures, there is a clear disagreement concerning the role of phonological aspects vs. phonological awareness in dyslexia (Tallal, 1980; Bradley and Bryant, 1983; Lyon, 1998; Stanovich, 2000; Snowling, 2001; Ramus et al., 2003). Livingstone et al. (1991) and Best and Demb (1999) have claimed that reading difficulties are due to a weakness of the magnocellular visual system. Bakker “Balance Model” (1992) describes dyslexia in terms of the alternation of hemispheric dominance. According to this model, normal reading is achieved by a balance between two distinct processes—-a first phase of visuo-perceptive analysis of the word (*via* the right cerebral hemisphere) and a second linguistic analysis phase (left hemisphere). The model suggests that, in people with dyslexia, there is an imbalance between the two hemispheres. Using PET, Nicolson et al. (2001) found deficiencies or anomalies at the cerebellar level in children with dyslexia.

Conversely, theories that center the explanation for dyslexia on anomalies in visuo-spatial processing also show remarkable differences in their interpretative models (see Geiger and Lettvin, 1987; Legge et al., 2002; Facoetti et al., 2006 for visuospatial attention). While some authors suggest that ocular movements play a key role in dyslexia (e.g., Rayner, 1998; Biscaldi et al., 2000), others have integrated the concepts of metaphonology, ocular movements, and the crowding effect to explain dyslexia (Spinelli et al., 2002). Some researchers have also proposed letters span to play a role, and have reported correlations with reading speed (Chung, 2002; Legge et al., 2007). There has also been recent interest in attentive-executive aspects that may be involved in reading difficulties (Denckla and Cutting, 1999; Shaywitz and Shaywitz, 2008; Varvara et al., 2014).

It becomes apparent from the literature that there may be several “dyslexias” and not a single category that unifies many different subjects. The use of “Aristotelian particulars” could help to delate the theoretical conflicts; it would be appropriate to refer to “some dyslexics” instead of “the dyslexics” (Benso, 2010). From this perspective, increasing evidence supports multi-componential models; this evidence has been logical (since avoiding “absolute affirmations” does not necessary introduce contradictions) and empirical, with experimental results in favor of a functional reading architecture that involves multiple systems (Menghini et al., 2010; Benso et al., 2013; Varvara et al., 2014), including executive attention and expression in WMC (Engle and Kane, 2004).

There have been reports of improvements in reading after WMC stimulation (Klingberg et al., 2005; Dahlin, 2010; Loosli et al., 2011), after tachistoscopic stimulation (Tressoldi et al., 2003), and after sub-lexical and neuropsychological sets (Tressoldi and Vio, 2011). The ICT method applied to reading difficulties involves the use of all these procedures; it works on WMC (and the various executive and attentive functions) and on sublexical and lexical processes, using instruments such as tachistoscopes and turbo-reader. Benso et al. (2008) reported a 0.9 syllables/second increase in reading speed after just 4 months of cognitive training (instead of the natural annual increase of 0.3 syllables/second), which was tailored according to the participants' emotive-motivational state.

### Hypotheses

We conducted two experiments to examine the effect of ICT in children with a diagnosis of dyslexia. Reading skills were measured before treatment, after the treatment, and after a 4-months follow up. We hypothesized that children with dyslexia would show increases in speed and accuracy of reading following ICT.

In Experiment 1, we hypothesized that 7 months of ICT in children with developmental dyslexia would enhance reading skills, both in accuracy and speed, beyond the threshold of the annual normo-readers' increase (Tressoldi et al., 2001). ICT focused on both executive attention and reading modules using a tachistoscope.

In Experiment 2, we hypothesized that children with dyslexia who completed only 18 ICT sessions would experience larger increases in accuracy and speed of reading than a control group of children with dyslexia subjected only to compensatory tools and dispense/helping procedures. We also hypothesized that this improvement would be stable, even after a blank period (without any training) of 4 months.

## Experiment 1

In the first experiment, we evaluated the efficacy of a 7-month ICT on a sample of children with dyslexia. The aim of this experiment was to verify the actual increase in accuracy and speed of reading, measured in syllables per second (syll/s), after administration of the ICT. The speed of reading and accuracy were assessed before and after the treatment period.

### Materials and Methods

#### Participants

Twenty children (12 male and 8 female) aged 8–14 years (mean = 9 years; SD = 1.36 years), whose parents opted for a 7-month ICT training for reading disorder, were enrolled in Experiment 1. Participants were admitted to Alassio Salute Medical Center, Italy, and received diagnosis of developmental dyslexia. Children were dignosed with developmental dyslexia by the National Health System, according to the guidelines of the national Consensus Conference (2010). The enrolled children presented with at least two points below the norm among 6 parameters: speed and accuracy in word, non-word and text reading (in DDE-2 and MT tests; Sartori et al., 1995; Cornoldi et al., 1998, see following section Materials and Procedure), had no sensory problems, and had an IQ within the normal population range.

#### Materials and Procedure

We employed the MT test (Cornoldi et al., 1998) of narrative text reading and the battery for evaluation of evolutionary dyslexia DDE-2 (and dysorthography; Sartori et al., 1995) for words and non-words reading. These assessment tools use Italian language and standardization, and are officially accredited and employed for the diagnosis of developmental dyslexia.

The rehabilitation protocol, explained in section ICT: Exercises and Materials, included specific exercises that stimulate the reading module with the aid of computer software, increase executive functions (Benso, 2004a, 2010, 2018), and engage language and visual-perception systems. The individual training sessions lasted 45 min and took place 2 days a week with a specialized operator. In addition, participants completed tachistoscope tasks and completed the APE 10 at home three times a week (for about 20/30 min), with the help of their parents. The treatments were administered during the months of school attendance. The experiment compared pre- and post-treatment reading skills within the same clinical group. The time interval between pre- and post-treatment assessments was 7 months. Parents provided written informed consent for their child's participation.

### Results

The analyses were performed using SPSS statistical software version 20.0. Raw and standard scores were analyzed. Standard Z-scores were used to rule out the effect of age covariance, since participants were in different school grades. For the “errors” parameter, and since the MT test provides a qualitative report, performance ranges were converted into numerical values to obtain quantitative data. The MT results were scored as follows: Request for Immediate Intervention = 0; Warning Request = 1; Sufficient Performance = 2; Criterion Completely Reached = 3. The children's IQ ranged from 30 to 75, with a mean value of 52.7 ± 14.3. The numerosity for each school grade and reading speed increase were: 2nd Grade (7 years old) *n* = 6, 1.36 syll/s; 3rd Grade *n* = 8, 1.13 syll/s; 4th Grade *n* = 1, 0.36 syll/s; 5th Grade *n* = 3, 0.86 syll/s; 6th Grade (11 years old) *n* = 1, 0.64 syll/s; 7th Grade *n* = 1, 0.96 syll/s. No statistical significative difference was found between QI (Raven Matrices expressed in percentiles) and speed increase of reading: *N* = 20, Spearman's rho = 0.022, *p* = 0.926.

After verifying the significance of the Kolmogorov-Smirnov test and that skewness and kurtosis values were not between 1 and −1, all dependent variables were deemed to be normally distributed (reading speed of text: Kolmogorov-Smirnov *p* = 0.493, skewness = 0.233, kurtosis = −0.346; Text errors: Kolmogorov-Smirnov *p* = 0.731, skewness = 0.203, kurtosis = −0.308; Reading speed of words: Kolmogorov-Smirnov p = 0.539, skewness = 0.651, kurtosis = 0.163; Errors of words: Kolmogorov-Smirnov *p* = 1.026, skewness = 0.901, kurtosis = 2.703; Reading speed of non-words: Kolmogorov-Smirnov *p* = 0.446, skewness = −0.169, kurtosis = −0.878; Errors of non-words: Kolmogorov-Smirnov *p* = 0.759, skewness = 0.054, kurtosis = −0.702; Analyses of standard scores showed similar values). However, given the small sample size, non-parametric tests were executed and reported along with parametric tests.

Finally, the effect size representing the strength with which a phenomenon is present or how much the null hypothesis is not accepted was calculated using Cohen's guidelines, whereby *r* < 0.10 negligible, 0.10 < *r* < 0.30 small, 0.30 < *r* < 0.50 moderate, *r* > 0.50 large; and *d* < 0.20 negligible, 0.20 < *d* < 0.50 small, 0.50 < *d* < 0.80 moderate, *d* > 0.80 large (Cohen, 1988).

The dependent variables were as follows: Reading speed (syll/s), reading accuracy (number of errors), for the conditions text reading, words and non-words reading. The observed raw values (means and SD) of the dependent variables and the corresponding Z-scores (weighted for school grade) measured at pre- and post-treatment are reported in Tables 1, 2, respectively.

**Table 1:** Exp 1: Reading raw scores (mean and standard deviation) pre and post treatment; errors number, and syllables per second (syll/s) of text reading MT, words reading DDE-2, and non-words reading DDE-2.

	**Text reading syll/s**	**Text reading errors**	**Words reading syll/s**	**Errors reading words**	**Non-words reading syll/s**	**Non-words reading errors**
Mean pre	1.49	13.35	1.21	11.50	1.06	13.25
SD pre	0.49	2.78	0.49	3.40	0.32	2.84
Mean post	2.57	7.45	2.09	7.35	1.62	7.30
SD post	0.53	2.84	0.48	2.68	0.34	1.95
Improvement syll/s	1.08		0.88		0.57	

**Table 2:** Exp 1: Average, minimum value (Min) and maximum value (Max) calculated on Z-scores; errors and syllables per second (syll/s) calculated on Z-scores of readings tests (text, words and non-words).

	**Z-scores text reading syll/s**	**Text Reading errors performance**	**Z-scores words reading syll/s**	**Z scores words reading errors**	**Z-scores non-words reading syll/s**	**Z-scores non-words reading errors**
Mean pre	−1.42	0.65	−1.69	1.64	−1.03	1.69
Min	−2.57	0	−2.98	0	−1.85	0.4
Max pre	−0.13	2	0.99	3.33	0.13	4.67
Mean post	−0.79	1.55	−1.10	1.51	−0.27	0.64
Min	−2.23	0	−2.87	−0.25	−1.67	−0.50
Max post	0.62	2	0.41	3.33	1.48	2.00
Improvement pre post	+0.63	+0.9	+0.59	−0.13	+0.76	−1.05

**Figure 1:**
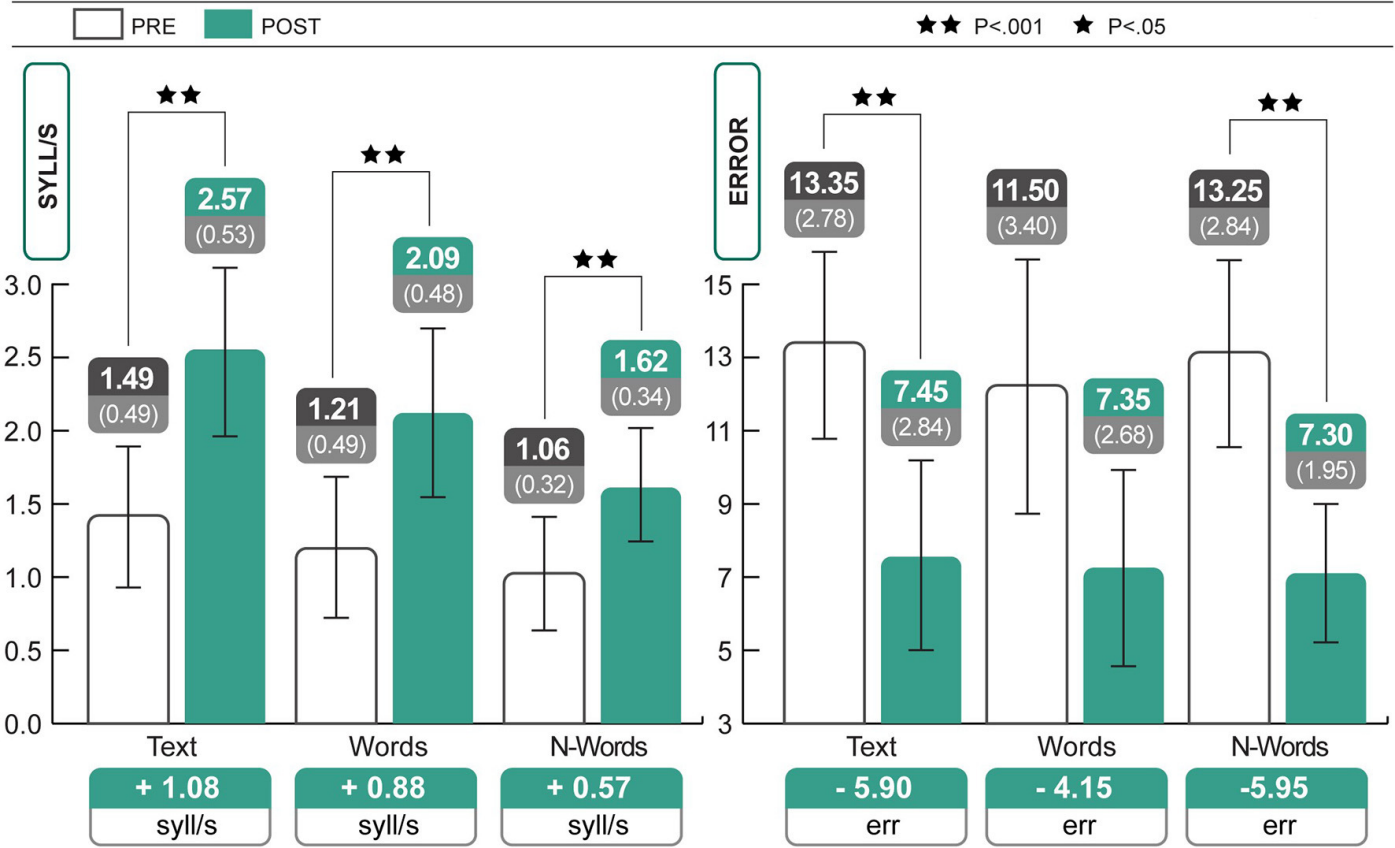
Significant increases in speed (text, words, and non-words) and accuracy (text and non-words) of reading. Statistical differences are marked according to the *p*-values at both Wilcoxon test and *t*-test on Z scores. The change in text reading speed was 494.4% higher than expected (+1.08 syll/s; see Discussion).

As seen in Table 1, subjects had gained in average 1.08 syll/s in the text test, 0.88 syll/s in the word test DDE-2, and 0.57 syll/s in the non-word test DDE-2 after training; mistakes also decreased. Statistical analyses of standardized values were carried out to verify the actual improvement, given that the subjects were from different grades. All test scores (see Table 1) showed significant improvements between pre and post-treatment periods (Wilcoxon test: reading speed of text: *Z* = −3.58; *p* < 0.001, *r* = 0.56; reading speed of words: *Z* = −3.59; *p* < 0.001, *r* = 0.57; reading speed of non-words: *Z* = −3.21; *p* < 0.001, *r* = 0.51; reading accuracy of text: *Z* = 3.22; *p* < 0.001; *r* = 0.58; reading accuracy of non-words: *Z* = −3.55; *p* < 0.001, *r* = 0.56). Analyses of the raw scores revealed the same results as those obtained using the Wilcoxon test (reading speed of text: *Z* = −3.92; *p* < 0.001; reading speed of words: *Z* = −3.92; *p* < 0.001; reading speed of non-words: *Z* = −3.85; *p* < 0.001; reading accuracy of text: *Z* = −3.94; *p* < 0.001; reading accuracy of words: *Z* = −3.80; *p* < 0.001; reading accuracy of non-words: *Z* = −3.87; *p* < 0.001). All the effect sizes (r) were large, according to Cohen's classification. Figure 1 shows the changes in reading speed and accuracy after 7 months of treatment.

We also verified the results using a parametric test, and therefore took advantage of the robustness of the *t*-test. *T*-test analyses of standard scores (Z scores) showed improvements in the reading speed of text [mean pre = −1.42, mean post = −0.79: *t*_(19)_ = −5.98, *p* < 0.001, *d* = 1.30], words [mean pre = −1.69, mean post = −1.10: *t*_(19)_ = −5.07, *p* < 0.001, *d* = 1.08], and non-words [mean pre = −1.03, mean post = −0.27: *t*_(19)_ = −4.05, *p* = 0.001, *d* = 0.88], and also revealed improvements in the reading accuracy of text [mean pre = 2.35, mean post = 1.45: *t*_(19)_ = −4.72, *p* < 0.001, *d* = 1.03]. There were no significant differences in reading accuracy of words [mean pre = 1.64, mean post = 1.51: *t*_(19)_ = 0.713, *p* = 0.485] and non-words [mean pre = 1.69, mean post = 0.64: *t*_(19)_ = 4.91, *p* < 0.001, *d* = 1.06]^3^.

To examine possible differences associated with disease severity, we also analyzed the results by splitting participants into mild and moderate dyslexia groups. The mild dyslexia group comprised 13 children with a −2 < Z-score < −1.5 in at least two parameters among speed and accuracy in the three tasks for text, words, and non-words reading. The moderate dyslexia group included 7 children with −3 < Z-score ≤ −2, in at least two of the above-cited parameters. None of the children had severe dyslexia.

A Mann-Whitney test revealed no significant difference between moderate and mild dyslexia groups in the pre-post training comparisons on reading speed (syll/s post- minus pre-training) of text (mean moderate = 0.96 syll/s, mean mild = 1.31, *Z* = −1.625, *p* = ns), words (mean moderate = 0.79 syll/s, mean mild = 1.06, *Z* = −1.665, *p* = ns), or non-words (mean moderate = 0.56 syll/s, mean mild = 0.57, *Z* = −0.227, *p* = ns). No significant between-group differences were found in reading accuracy (number of errors, post minus pre) of text (mean moderate = −5.77, mean mild = −6.14, *Z* = −0.36, *p* = ns), words (mean moderate = −3.38, mean mild = −5.57, *Z* = −1.25, *p* = ns), or non-words (mean moderate = −5.62, mean mild = −6.57, *Z* = −0.64, *p* = ns).

### Discussion

The association between developmental rate of reading and physiological changes has been reported by Tressoldi et al. (2001). Such a rate represents a benchmark to establish the “goodness” of a rehabilitative treatment of reading. Tressoldi et al. found that the annual natural growth rate of reading speed in Italian children with dyslexia is around 0.3 syll/s for words and text (against 0.5 syll/s of normo-readers) and 0.14 syll/s for non-words (against 0.28 syll/s of normo-readers). Using this information, we calculated that a dyslexic subject should gain 0.18 syll/s over 7 months (0.3 syll/s for normo-readers). Therefore, the expected 7-month improvement for children with dyslexia was 0.18 syll/s; however, we found an improvement of 1.08 syll/s after 7 months of ICT, which exceeds the natural improvement expected in normo-readers over 1 year. On average, the change was 494.4% higher than the expected rate. Moreover, accuracy also improved in all participants after ICT. The lack of a control group is one limitation of the experiment; however, participants exceeded the normo-readers' speed increase rate and statistical analyses were applied to Z-scores, which indicates that ICT improved reading in children with dyslexia beyond the rate expected in the general population. The mean differences revealed larger improvements in the mild dyslexia group than in the moderate dyslexia group, but this difference was not significant; this could be due to sample size limitations. It is possible that a more mature “lexical” reading strategy was used by the children with mild dyslexia, which could promote reading improvement. Further studies should investigate this hypothesis.

However, it would be interesting to obtain a comparative measure of the normal developmental changes in children with dyslexia who are not subjected to a specific training, and, moreover, to examine whether the beneficial changes in reading due to ICT remains stable over time. In Experiment 2, we addressed these issues.

## Experiment 2

In Experiment 1, we found a remarkable improvement in reading speed and accuracy in children with dyslexia after a 7-month period of ICT treatment administered in bi-weekly 45-min sessions. To verify that this improvement was not a result of normal developmental factors and to examine the stability of this change months after the training, a second study was conducted. In Experiment 2, children with reading disorders were allocated to either the experimental group and subjected to the ICT treatment, or to the control group and subjected to no specific cognitive training. The control group did, however, have access to the compensatory tools and helping procedures provided by the scholastic Personalized Educational Plan. To evaluate the maintenance of improvement after ICT, a retest phase was subsequently performed on a sub-group of the experimental group 4 months after the training. To increase the number of weekly sessions, parents were also trained to execute specific ICT home exercises.

### Materials and Methods

#### Participants

Twenty-six participants, aged between 8 and 14 years who had been diagnosed with developmental dyslexia were recruited. Children had been diagnosed with developmental dyslexia by the National Health System, according to the guidelines of the national Consensus Conference (2010). Participants were allocated into two groups of 13 participants each, including the experimental group, which was subjected to the ICT treatment, and the control group, which received no specific cognitive training. The two groups were matched by age (experimental group mean = 10.92 years, SD = 1.19 years; control group mean = 10.85 years, SD = 1.21 years; the difference was not significant, Mann Whitney test; *U* = 77.5, *Z* = −0.37, *p* = 0.710), sex (7 male and 6 female participants in both groups), IQ (*p* > 0.05, Table 3), and disorder profile. Parents provided signed informed consent for their child's participation in the study.

**Table 3:** Scores of the two groups in T0 (pre training phase): number of subjects (N), mean (M), minimum value (Min) and maximum value (Max), standard deviation (SD) calculated on raw and Z-scores.

**Comparisons between the two groups at T0**										
	**Control group**				**Experimental group**				**Mann-Whitney test**	
	***N***	**M**	**Min Max**	**SD**	***N***	**M**	**Min Max**	**SD**	***Z***	***p***
IQ Intelligence Quotient	13	100.31	85 110	8.26	13	102.77	87 120	10.35	−0.85	0.395
Text reading MT syll/s	13	1.92	0.37 3.84	1.18	13	2.43	1.23 3.87	0.76	−0.80	0.425
Z-scores text reading MT speed	13	−2.08	−4.0 −0.29	0.93	13	−1.61	−2.34 −0.68	0.47	−1.09	0.913
Text reading errors	13	12.38	3 24	5.94	13	11.23	1 23	6.42	−1.25	0.209
Text reading errors performance slot	13	2.08	1 4	1.04	13	2.15	1 4	1.21	−1.62	0.106
Non-words reading syll/s	13	1.29	0.50 2.59	0.63	13	1.36	0.66 2.44	0.56	−0.28	0.778
Non-words reading speed Z-scores	13	−1.53	−2.4 −0.35	0.75	13	−1.46	−2.23 −0.32	0.65	−0.48	0.626
Non-words reading errors	13	7.31	1 19	5.04	13	7.00	4 10	2.27	−0.39	0.698

*Comparisons between two groups analyzed by Mann-Whitney test for two independent samples: Z-value (Z) and p-value (p) of probability are reported*.

#### Materials and Procedure

The same ICT protocol as that used in Experiment 1 was applied to the experimental group. The treatment was administered from April (T0)^4^ to June (T1), for a total of 18 training sessions held twice a week. Each session lasted 45 min and was conducted by a trained operator. At the same time, tachistoscope and APE 10 exercises in sessions that lasted from 20 to 30 min were carried out at home three times a week with the help of the parents and under the operator's supervision. All participants in the experimental group carried out an obligatory sporting/artistic activity (minimum, twice a week) to support executive attention (Sakai et al., 2002; Seidler et al., 2012; Benso, 2018). The control group had access to compensatory tools and helping procedures provided by the scholastic Personalized Educational Plan.

The speed and accuracy of reading were evaluated before and after the treatment period using the MT test (Cornoldi et al., 1998) of narrative text reading and the battery for evaluation of evolutionary dyslexia DDE-2 (Sartori et al., 1995) for non-words^5^. The maintenance of the increase in reading skills were evaluated by re-administering the same MT and DDE-2 tests 4 months later, after the summer holidays (T2: October), a period during which participants did not receive any cognitive or reading training. Unfortunately, only 6 of the 13 participants in the experimental group were available for these follow-up T2 tests.

### Results

As in Experiment 1, the analyses were performed using SPSS 20.0, and both raw and standard scores were analyzed given that participants belonged to different school grades. For the “errors” parameter, performance ranges in the MT were converted into numerical values, as follows: Request for Immediate Intervention = 1; Warning Request = 2; Sufficient Performance = 3; Criterion Completely Reached = 4.

After verifying the significance of the Kolmogorov-Smirnov test and that the skewness and kurtosis values were not between 1 and −1, the data distributions were considered to be normally distributed (i.e., speed of reading text: Kolmogorov-Smirnov *p* = 0.680, skewness = 0.538, kurtosis = 0.250; other variables showed similar values). However, given the small sample size (*N* < 20), non-parametric tests were used to analyze the data.

Within-group comparisons of dependent variables were made using the Wilcoxon test (on raw data), and between-group analyses were carried out using Mann-Whitney tests for independent samples (mainly on standardized scores). Effect sizes were calculated, and Corbetta and Shulman (2002) guidelines were used, as follows: *r* < 0.10 negligible, 0.10 < *r* < 0.30 small, 0.30 < *r* < 0.50 moderate, *r* > 0.50 large. For the Mann-Whitney test, the rank biserial correlation (Glass, 1966) was also computed.

To assess the efficacy of ICT, a comparison was made with the estimated measure of natural change (without treatment) of the control group and the level achieved by the experimental group.

In the pre-test phase (T0) no significant between-group difference in reading skills was found using the Mann-Whitney test for two independent samples (all *p* > 0.05; see Table 3). There was a clear matching of reading text and non-words between the groups at T0.

**Figure 2:**
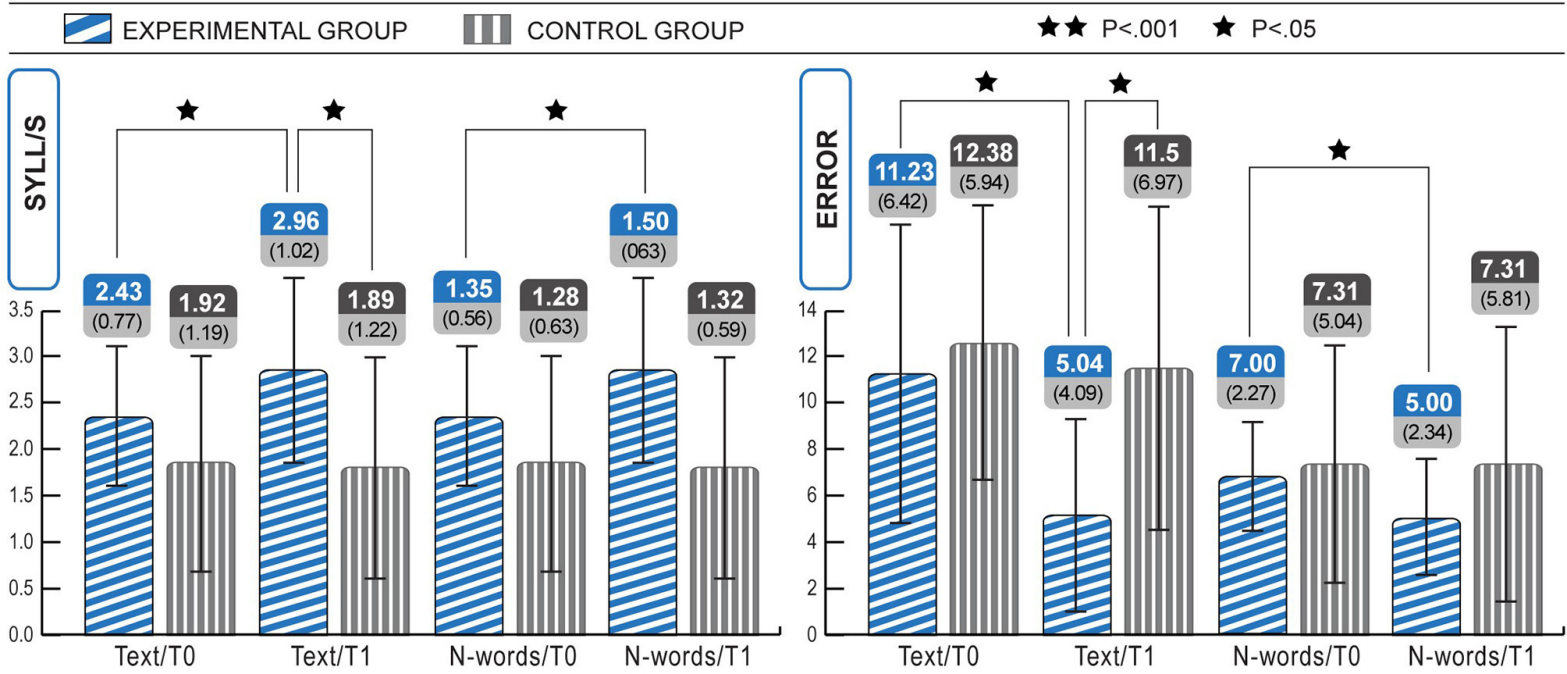
Within and between group analyses show significant increases in speed (text and non-words) and accuracy (text and non-words) of reading in the Experimental group, from T0 to T1. Statistical differences are marked according to the *p*-values at Wilcoxon test on Z scores. The change in speed text was 607% higher than expected in the experimental group.

The comparison between the experimental and control groups at T1 (see Table 4) using the Mann-Whitney test for two independent samples, revealed significant differences in both the speed (Z scores, mean control group = −2.16, mean experimental group = −1.31: *Z* = −2.51, *p* = 0.012) and accuracy (mean control group = 2.00, mean experimental group = 3.15: *Z* = −2.58, *p* = 0.010) of text reading. The differences had a large effect size (r) in the MT text reading speed on standard scores (*r* = 0.49) and MT text reading error performance (*r* = 0.51). There were no significant differences in the two parameters of non-word reading. See Figure 2 for pre-post comparisons of the raw data^6^.

**Table 4:** Comparisons of two independent groups at T1; values are expressed in raw and Z-scores; errors are codified in agreement with MT guidelines (1: request for immediate intervention; 2: warning request; 3: sufficient performance; 4: criterion completely reached).

**Comparisons of two groups at T1 (Retest 1)**.								
	**Control group**		**Experimental group**		**Mann-Whitney test**			
	**M**	**SD**	**M**	**SD**	***Z***	***p***	***r***	**CRB**
Text reading MT Z-score	−2.16	0.95	−1.31	0.53	−2.51	0.012	0.49	0.58
Text reading errors performance	2.00	1.08	3.15	0.89	−2.58	0.010	0.51	0.57
Non-words reading syll/s Z-score	−1.46	0.70	−1.23	0.69	−0.95	0.342		
Non-words reading errors Z-scores	0.75	1.47	0.20	0.71	−0.85	0.395		

*Comparisons are calculated on Mann-Whitney test: Z-value (Z), p-values (p) of probability, effect size (r and CRB) are reported*.

As shown in Table 5 and Figure 2, the experimental group gained about 0.5 syll/s in text reading after 18 ICT sessions, and the error rate decreased remarkably. Indeed, rate and accuracy significantly improved for both text and non-word reading, and with a large or moderate effect size (r). The Wilcoxon test for two dependent samples showed improvements in MT text reading speed on Z-scores (mean T0 = −1.61, mean T1 = −1.31; *Z* = −2.41; *p* < 0.05, *r* = 0.47), MT text reading error performance (rating from 1 to 4, mean T0 = 2.12, mean T1 = 3.15; *Z* = −2.23; *p* < 0.05, *r* = 0.64), non-word reading syll/s (mean T0 = 1.35, mean T1 = 1.50; *Z* = −2.77; *p* < 0.05, *r* = 0.44), non-word reading errors (mean T0 =0.83, mean T1 = 0.19; *Z* = −2.04; *p* < 0.05, *r* = 0.40).

**Table 5:** Experimental group, comparisons within group, calculated on Wilcoxon test for two dependents groups; number of subjects (*N*), mean pre and post training (M T0 and M T1), standard deviation (SD), *Z*-value of Wilcoxon (*Z*), *p*-value (*p*), effect size (*r*).

**Experimental group comparisons within group (T0); T1 (Retest 1) at the Wilcoxon test**.							
	**M T0**	**M T1**	**SD T0**	**SD T1**	***Z***	***p***	***r***
Text reading MT syll/s	2.43	2.96	0.77	1.02	−3.18	0.001	**0.62**
Text reading MT Z-score	−1.61	−1.31	0.46	0.53	−2.41	0.016	**0.47**
Text reading MT errors	11.23	5.04	6.42	4.09	−2.77	0.006	**0.54**
Text reading errors performance slot	2.12	3.15	1.11	0.90	−2.23	0.026	**0.64**
Non-words reading syll/s	1.35	1.50	0.56	0.63	−2.27	0.023	**0.45**
Non-words reading syll/s Z-score	−1.46	−1.22	0.65	0.69	−2.27	0.023	**0.44**
Non-words reading errors	7.00	5.00	2.27	2.34	−2.00	0.045	**0.41**
Non-words reading errors Z-scores	0.83	0.19	0.71	0.71	−2.04	0.041	**0.40**

The control group showed no increases between pre- and post-treatment evaluation (all *p* > 0.05; see Table 6). Namely, there was no within-group difference in MT text reading speed on standard scores (mean T0 = −2.08, mean T1 = −2.16; *Z* = −0.94; *p* = ns), MT text reading error performance (mean T0 = 2.08, mean T1 = 2.00; *Z* = −0.58; *p* = ns), non-word reading syll/s standard score (mean T0 = −1.52, mean T1 = −1.46; *Z* = −1.07; *p* = ns), non-word reading errors standard score (mean T0 = 0.76, mean T1 = 0.75; *Z* = −0.14; *p* = ns).

**Table 6:** Control group, comparisons within group, calculated on Wilcoxon test for two dependents groups; number of subjects (*N*), mean pre and post training (M T0 and M T1), standard deviation (SD), *Z*-value of Wilcoxon (*Z*), *p*-value (*p*), effect size (*r*).

**Control groups comparisons within groups between T0 (pre) T1 (post): Wilcoxon test**							
	**M T0**	**M T1**	**SD T0**	**SD T1post**	***Z***	***p***	***r***
Text reading MT syll/s	1.92	1.89	1.19	1.22	−0.28	0.78	
Text reading MT Z-score	−2.08	−2.16	0.93	0.95	−0.94	0.345	
Text reading MT errors	12.38	11.50	5.94	6.97	−0.94	0.348	
Text reading errors performance slot	2.08	2.00	1.03	1.08	−0.58	0.564	
Non-words reading syll/s	1.28	1.32	0.63	0.59	−1.11	0.266	
Non-words reading syll/s Z-score	−1.52	−1.46	0.75	0.70	−1.07	0.286	
Non-words reading errors	7.31	7.31	5.04	5.81	−2.11	0.833	
Non-words reading errors Z-scores	0.76	0.75	1.31	1.47	−0.14	0.889	

#### Retest 2

**Figure 3:**
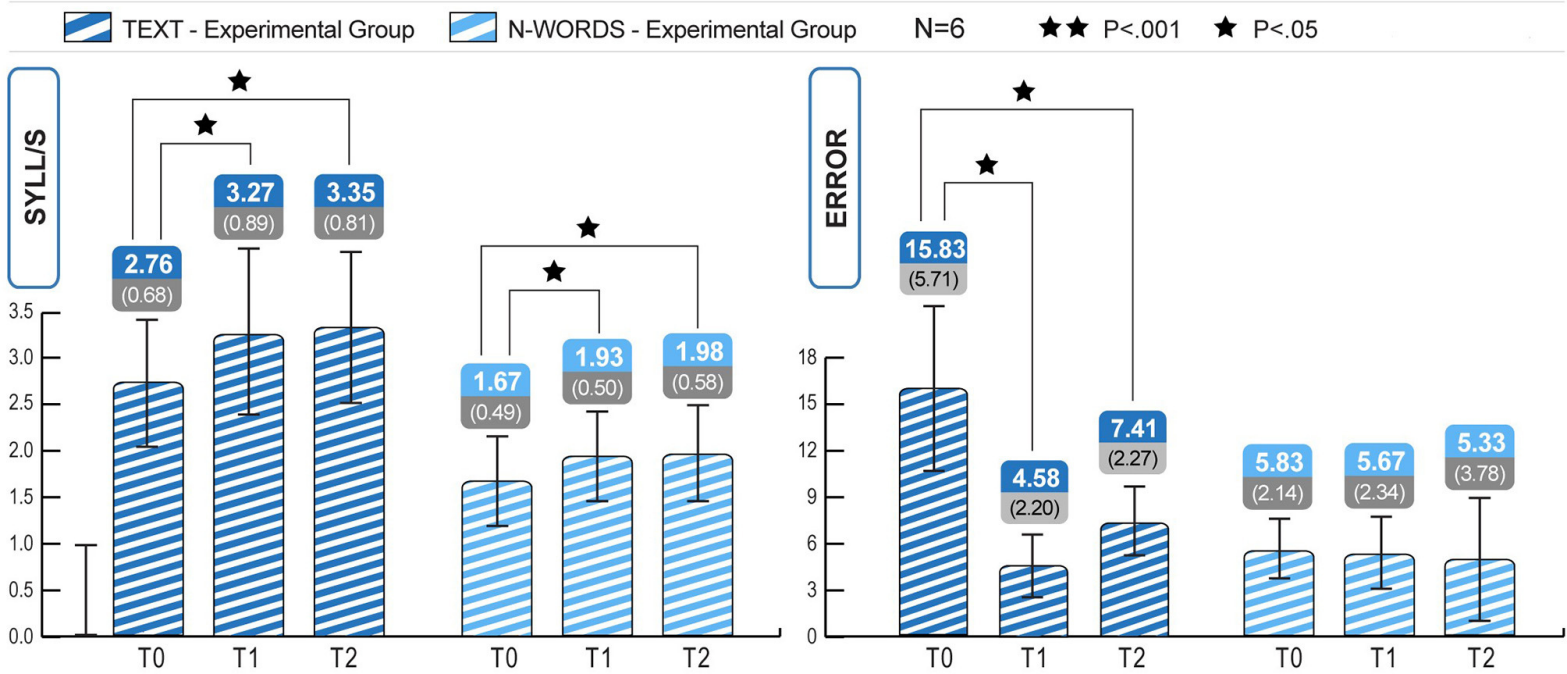
Experimental group, within subjects comparisons. Significant increase in text speed and accuracy from T0 to T1 and from T0 to T2 and in non-words speed from T0 to T1 and from T0 to T2 (Tables 5, 7). Statistical differences are marked according to the *p*-values at Wilcoxon test on Z scores. No significant change of the improvement obtained in T1, compared to the measures at T2 (all *p* > 0.05).

To verify the robustness of the ICT-induced improvements, reading skills were retested 4 months after the end of treatment (T2 phase) in 6 participants in the experimental group. The longitudinal study of the three phases T0, T1, T2 clarified the ICT-induced improvement trend and its retention after 4 months. As seen in Figure 3, the Wilcoxon test revealed significant differences between T0 and T1 in text reading speed (mean T0 = 2.76, mean T1 = 3.27, *Z* = −2.207; *p* < 0.05), text reading accuracy (mean T0 = 15.83, mean T1 = 4.58, *Z* = −2.21; *p* < 0.05), and non-word reading speed (mean T0 = 1.67, mean T1 = 1.93, *Z* = −2.20; *p* < 0.05). The Wilcoxon test results (see Tables 7, 8) revealed that a significant increase in reading speed and accuracy was still present between T0 and T2, and with a large effect size (r). This was true for the speed of text reading (mean T0 = 2.76, mean T2 = 3.35, *Z* = −2.20; *p* = 0.28; *r* = 0.64) and accuracy of text reading (mean T0 = 15.83, mean T2 = 7.41, *Z* = −2.70; *p* = 0.38; *r* = 0.59), and for the speed of non-word reading (mean T0 = 1.67, mean T2 = 1.98, *Z* = −2.207; *p* = 0.27; *r* = 0.64).

**Table 7:** Experimental group, comparisons within group (N.6), calculated on Wilcoxon test for two dependent groups; number of subjects (*N*), mean pre and post training (M T0 and M T2), standard deviation (SD), *Z*-value of Wilcoxon (*Z*), *p*-value (*p*), effect size (*r*).

**Experimental group comparisons within group T0 vs. T2: Wilcoxon test**									
	***N***	**M T0**	**M T2**	**SD T0**	**SD T2**	***Z***	***p***	***r***
Text reading MT syll/s	6	2.76	3.35	0.68	0.81	−2.201	0.028	0.64
Text reading MT errors	6	15.83	7.41	5.71	2.27	−2.070	0.038	0.59
Non-words reading syll/s	6	1.67	1.98	0.49	0.58	−2.207	0.027	0.64
Non-words reading errors	6	5.83	5.33	2.14	3.78	−0.365	0.715	

**Table 8:** Experimental group, comparisons within group, calculated on Wilcoxon test for two dependents groups; number of subjects (*N*), mean pre and post training (M T1 and M T2), standard deviation (SD), *Z*-value of Wilcoxon (*Z*), *p*-value (*p*), effect size (*r*).

**Experimental group; comparisons within group T1 vs. T2, Wilcoxon test**								
	***N***	**M T1**	**M T2**	**SD T1**	**SD T2**	***Z***	***p***	***r***
Text reading MT syll/s	6	3.27	3.35	0.89	0.81	−1.09	0.273	
Text reading MT Z-score	6	−1.17	−1.31	0.49	0.34	−1.36	0.173	
Text reading MT errors	6	4.58	7.41	2.20	2.27	−1.46	0.144	
Text reading errors performance	6	3.33	2.67	0.52	0.52	−1.63	0.102	
Non-words reading syll/s	6	1.93	1.97	0.50	0.58	−1.10	0.269	
Non-words reading syll/s Z-score	6	−0.66	−0.97	0.46	0.59	−1.57	0.116	
Non-words reading errors	6	5.67	5.33	2.34	3.78	−0.28	0.783	
Non-words reading errors Z-scores	6	0.53	0.28	0.69	0.85	−0.41	0.680	

The Wilcoxon test analyses, reported in Table 8, revealed that the improvements obtained at T1 were stable after 4 months of treatment suspension (T2). As shown in Figure 3, the results obtained at T1 (retest 1 phase) were maintained over time in the experimental group, and in the absence of direct stimulation of the compromised modules; there were no significant differences between raw and standardized data (Table 8). The *p*-values for every assessment trial were >0.05. Furthermore, the Wilcoxon test performed using standard scores revealed no significant differences in MT reading speed (mean T1 = −1.17, mean T2 = −1.31, *Z* = −1.36, *p* = ns), reading accuracy (mean T1 = 3.33, mean T2 = 2.67, *Z* = −1.63, *p* = ns), non-word reading speed (mean T1 = −0.66, mean T2 = −0.97, *Z* = −1.57, *p* = ns), or non-word reading accuracy (mean T1 = 0.53, mean T2 = 0.28, *Z* = −0.41, *p* = ns). Thus, the subjects retested in October (T2) retained reading skills that had been achieved at the end of treatment (June, i.e., T1). The Wilcoxon test performed using raw data did not reveal improvements in MT reading speed (mean T1 = 3.27, Mean T2 = 3.35, *Z* = −1.09, *p* = ns), MT reading accuracy (mean T1 = 4.58, mean T2 = 7.41, *Z* = −1.43, *p* = ns), non-word reading speed (mean T1 = 1.93, Mean T2 = 1.97, *Z* = −1–10, *p* = ns), or non-word reading accuracy (mean T1 = 5.67, mean T2 = 5.33, *Z* = −0.28, *p* = ns). Figure 3 shows the number of errors, the syllables per second, and the progress maintained after 4 months.

### Discussion

The results of Experiment 2 demonstrate the efficacy of ICT. We found that ICT was associated with a gain of about 0.5 syll/s after 18 sessions in children with dyslexia, and this effect was stable in a group of 6 experimental participants evaluated 4 months after treatment. Participants in the control group that had access to the scholastic Personalized Educational Plan did not exhibit any appreciable changes in either reading speed or accuracy.

The data of Tressoldi et al. (2001) on the normal syll/s gain over 3 months indicate that a dyslexic subject would gain 0.075 syll/s of text reading, while this would be 0.125 syll/s in a normo-reader. Therefore, while the expected improvement would be 0.075 syll/s over the course of this study, children who received ICT achieved an improvement of 0.53 syll/s, which represents an average change of 607% higher than expected in the experimental group. Moreover, 4 months after the end of ICT, which is a period longer than the training itself, the acquisitions were still present and stable.

## General Discussion and Conclusions

We hypothesized that the multi-component ICT on the reading module would be effective. This hypothesis was supported by the significant improvements exhibited by participants in Experiment 1 (1.08 syll/s in text reading after 7 months) and by the experimental group in Experiment 2. Experiment 1 showed that ICT was associated with a significant improvement of both reading speed and accuracy. In Experiment 1, the assessment tools revealed a significant improvement between pre- and post-treatment phases in the reading speed of text, words, and non-words, and in reading accuracy of text and non-words.

In Experiment 2, the experimental group showed significant improvements in both speed and accuracy for text and non-word reading between pre-training T0 and retest T1. The effect sizes (r, d, rank biserial correlation CRB) were “large,” according to Corbetta and Shulman (2002). The same comparison for the control group revealed no significant improvement in any parameter. In the same period (from T0 to T1), a comparison between the experimental and control groups (Retest 1) showed that the experimental group performed significantly better in both speed and accuracy. In the children who received ICT, there was an improvement in text reading speed that was maintained even after a 4-month break. The parameter of accuracy improved for text reading and remained stable for non-words.

Despite the positive results of the ICT, a longer treatment period is recommended (considering that of Experiment 2). As an alternative, we could suggest more cycles of the same duration, with a possible break between them. These studies are necessary to further support the efficacy of the ICT methodology that we have shared in the present paper, which is based on research spanning over more than two decades. At the same time, in light of new advances in neuroscience, the ICT protocol has been improved upon over time, and has been found to be effective in various areas, such as people with dyslexia (Benso et al., 2008), in patients with mild cognitive impairment as assessed using PET (Ciarmiello et al., 2015; see also Rueda et al., 2005), and in young tennis players (Benso et al., 2018), and the ICT protocol has also been integrated into pedagogical methods that aim to improve academic achievement (Veneroso et al., 2018).

Therefore, we ascribe the results described in this work to the fact that stimulation was applied to both the modular part and the attentive-executive system (including WM), and avoided stereotyped and automatic modes of administration, thus ensuring that the training was “adaptive” (Metzler-Baddeley et al., 2016). Attentive pre-activation exercises (which add value to any kind of training or survey) and the elicitation of particular attentional states were carried out by a skilled operator. Work from Sarter et al. (2006) has highlighted the relevance of inducing a particular mental and neurobiological state that corresponds to “attentional effort.” Time duration in this state is mediated by the cholinergic circuits of the prefrontal/anterior cingulate and mesolimbic regions, and represents a top-down behavioral control by modulation of motivation, reward prediction, error monitoring, and sustained attention.

The ICT operator's characteristics are also relevant. The operator should have a theoretical understanding of neurosciences models of executive attention and working memory, the ability to develop a therapeutic “alliance,” and an empathic and motivating relationship. Moreover, the operator should supervise the rehabilitative setting and coordinate help provided by parents, teachers, and coaches. An empowering training on mindfulness protocols (recently included in ICT) would be appreciable. Furthermore, the attentional networks (alert, orienting, the SN, CEN, and DMN) that we consider in the present study have been theorized to be involved in the cyclical phases of mindfulness (e.g., Malinowski, 2013). We suggest that ICT operators (clinicians, sportive trainers, and teachers) practice dynamic mindfulness by themselves before applying this method to those receiving ICT. The “dynamic” version of ICT is particularly suitable to those who are of a developmental age and to hyperactive children. The aim is to induce more static and controlled phases later on in the treatment, when the self-regulating systems that are underpinned by the neural networks described above become reinforced.

With this in mind, it should be noted that each child in the study benefited from a treatment that was tailored to his/her specific needs (adaptivity) and that the selected operators complied with the essential criteria of the ICT protocol. Therefore, the experimental results of skills improvement/acquisition and their stability over time offer additional data in favor of the ICT efficacy. Previous work has proposed that learning stabilization is obtained and reinforced by the stimulation of isolated neural circuits (e.g., Wagner et al., 1998; D'Esposito et al., 2000; Menon and Uddin, 2010) and is regarded as essential for memorization in the learning phase. We conclude by pointing out the relevance of ensuring further appropriate qualifications to promote the attainment of autonomy in lacking skills, which the use of only dispensation and compensatory scholastic instruments cannot provide, even though they are useful.

For the sake of completeness, it is important to underline that after strengthening the attentive-executive system, participants reported being able to maintain longer and more intense study time (compared with baseline), to improve planning and organization skills in everyday life and, as far as self-perception is concerned, family members reported an increase in self-esteem and perceived self-efficacy. We can suggest that these results are due both to the targeted training of weaknesses and to the enhancement of the method of study that emerges during the course of ICT.

### Limitations and Further Research

Limitations of this work primarily concern experimental elements rather than the cognitive training itself. Specifically, there was no age-matched control group in Experiment 1; however, the improvements observed exceeded those predicted by the normal developmental rate, and pre-post significant differences were calculated on age-standardized Z scores.

One limitation of Experiment 2 is the small number of participants who completed the Retest 2 phase (T2), which was due to a lack of availability. However, the conclusion of a maintenance of reading levels acquired after ICT treatment is supported by a binomial distribution made on the minimum number of 6 subjects. In the experimental group, no children exhibited a decrease in the reading rate or accuracy. Hence, if we consider *decrease/not decrease* as a dichotomous variable, we have the probability of 1/2. To evaluate the probability that an event that has a *p*-value of 0.5 repeats itself 6 times in succession in the same way, we can use the formula of the binomial calculus (see below). The results was *p* = 0.0156, and this significance would be the probability that such event is due to the case.

$$\begin{array}{l} P\;\left(X=x\right)=\;\left(\begin{array}{c} n\\ x \end{array}\right)\;p^{x\;}q^{n-x};\;P\;\left(X=0\right)=\;\left(\begin{array}{c} 6\\ 0 \end{array}\right)\;\times \;0.5^{0}\;\times \\ {0.5\;}^{6-0}=1\;\times \;1\;\times \;0.0156=\;0.0156 \end{array}$$

Furthermore, for both experiments, considering the executive attention treatments and WM training, it would have been interesting to measure any increases in these skills, as has been reported in our previous work. Considering the sample size, possible influence of differences in socio-demographic and educational levels on ICT outcomes has not been evaluated and can hence be addressed in new experimental protocols.

In the future, it will be necessary to replicate this work with more consistent experimental groups and controls, which would also enable subgroup analyses and elucidate the involvement of specific learning and executive attention systems. Furthermore, inspired by various protocols for sports activities that we have developed in collaboration with researchers in motor science, and given the importance of motivational and emotional systems, we would like to evaluate developmental indices both in the assessment and treatment phases. For example, future work could evaluate stress values in addition to cognitive measurement and treatment (especially in the pre-treatment phase) using physiological indicators (for example heart rate variability, e.g., Gazzellini et al., 2017; or stress sensors based on galvanic skin responses, e.g., Vabbina et al., 2015). Furthermore, during the phases in which the subject is trained to maintain an attentional state over time in a state of tonic alertness (Sadaghiani et al., 2010), it would be interesting to record QEEG to obtain psychophysiological independent evidence of mental states. For this, we could partially replicate the measures of DMN intrusion in task-oriented activity observed with pediatric patients with traumatic brain injury (Gazzellini et al., 2016).

We are confident that by applying the principles highlighted to construct a more effective training protocol, we could apply the ICT to pathologies and conditions that have limited effective interventions.

## Data Availability Statement

The raw data supporting the conclusions of this article will be made available by the authors, without undue reservation.

## Ethics Statement

Ethical review and approval was not required for the study on human participants in accordance with the local legislation and institutional requirements. Written informed consent to participate in this study was provided by the participants' legal guardian/next of kin.

## Author Contributions

FB and SM conceived and designed the experiments. EB, SM, and VB performed the experiments. FB and EA analyzed the data. FB, SM, EB, EA, and SG contributed materials/analysis tool. FB, EB, SM, VB, EA, and SG contributed to the writing of the manuscript. All authors contributed to the article and approved the submitted version.

## Conflict of Interest

The authors declare that the research was conducted in the absence of any commercial or financial relationships that could be construed as a potential conflict of interest.

## Footnotes

^1^Among high-level athletes, the goal of coaches during training is “*to deconstruct the automatisms*”; see Turatto et al. (1999) for a study on the coaches of the national women's ski team.

^2^To carry out the initial “meditative” breathing phase, it is first necessary to create a therapeutic alliance, especially with hyperactive subjects. They are gradually led to accept the initial breathing phase, and will have less constraints and unnecessary impositions through more dynamic states of awareness and motor coordination. Subsequently, the association with temporal variations of the gesture is made, and, finally, the combination with breathing is achieved. This is already a sort of mindfulness, whereby thoughts are gradually centered, in a growing “here and now” state, which generates behavioral and cognitive benefits (e.g., Tang et al., 2012; Mrazek et al., 2013).

^3^Parametric Student's t-test analyses with repeated measures overlapped with the Wilcoxon test results presented. Cohen's d value revealed large effect sizes (*d* >0.80, see Cohen's guidelines, Corbetta and Shulman, 2002). The Student's t-test analyses on raw scores showed significant differences for reading speed of text [t_(19)_ = −11.91, *p* <0.001, *d* = 2.56], words [t_(19)_ = −12.15, *p* < 0.001, *d* = 2.55], and non-words [*t*_(19)_ = −7.68, *p* < 0.001, *d* = 1.65], and reading accuracy of text [*t*_(19)_ = 10.42, *p* < 0.001, *d* = 2.27], words [*t*_(19)_ =0.605, *p* < 0.001, *d* = 1.32], and non-words [*t*_(19)_ = 7.29, *p* < 0.001, *d* = 1.58 (Figure 1)].

^4^T0 is the pre-training phase (April); T1 is post-training phase (June); T2 is 4 months after T1 (October).

^5^Since short time, DDE-2 word reading test has not been employed and MT text reading and DDE-2 non-words reading have been used as representative of reading skills.

^6^A two-way ANOVA (Group X Condition, 2 x 2), with Group as the between factor and Condition (pre vs. post) as the within factor (mixed design), on Z-scores for text speed, revealed a significant main effect of group [Group: *F*_(1, 24)_ = 5.5, *p* < 0.05; Condition: *F*_(1, 24)_ = 1.8, *p* = ns]. The Condition X Group interaction was significant [*F*_(1, 24)_ = 4.9, *p* < 0.05]. *Post hoc* analyses revealed significant pre vs post training difference for the Experimental Group *t*_(12)_ = −2.92, *p* = 0.013, but not significant for the Control Group *t*_(12)_ = 0.546, *p* = ns. A two-way ANOVA on Z-scores of text accuracy was not carried out since this was a categorial variable. A mixed two-way ANOVA (Group X Condition, 2x2) on non-word Z-scores speed revealed no significant main effects of group or condition [Group: *F*_(1, 24)_ =0.36, *p* = ns; Condition: *F*_(1, 24)_ = 2.2, *p* = ns], and no condition by group interaction [*F*_(1, 24)_ = 0.84, *p* = ns]. The mixed two-way ANOVA (Group X Condition 2x2) on non-word Z-scores errors revealed no significant main effects [Group: *F*_(1, 24)_ = 0.39, *p* = ns; Condition: *F*_(1, 24)_ = 2.9, *p* = ns] and no Condition X Group interaction [*F*_(1, 24)_ = 2.6, *p* = ns].
